# Identifying biological landmarks using a novel cell measuring image analysis tool: Cell-o-Tape

**DOI:** 10.1186/1746-4811-8-7

**Published:** 2012-03-02

**Authors:** Andrew P French, Michael H Wilson, Kim Kenobi, Daniela Dietrich, Ute Voß, Susana Ubeda-Tomás, Tony P Pridmore, Darren M Wells

**Affiliations:** 1 Centre for Plant Integrative Biology, University of Nottingham, Sutton Bonington Campus, Nottingham LE12 5RD, UK

**Keywords:** Image analysis, Confocal, Arabidopsis, Software, Quantification

## Abstract

**Background:**

The ability to quantify the geometry of plant organs at the cellular scale can provide novel insights into their structural organization. Hitherto manual methods of measurement provide only very low throughput and subjective solutions, and often quantitative measurements are neglected in favour of a simple cell count.

**Results:**

We present a tool to count and measure individual neighbouring cells along a defined file in confocal laser scanning microscope images. The tool allows the user to extract this generic information in a flexible and intuitive manner, and builds on the raw data to detect a significant change in cell length along the file. This facility can be used, for example, to provide an estimate of the position of transition into the elongation zone of an Arabidopsis root, traditionally a location sensitive to the subjectivity of the experimenter.

**Conclusions:**

Cell-o-tape is shown to locate cell walls with a high degree of accuracy and estimate the location of the transition feature point in good agreement with human experts. The tool is an open source ImageJ/Fiji macro and is available online.

## Background

Software capable of aiding the manual phenotyping of plants has become the subject of much interest in recent times [1]. There has been a push to develop high throughput imaging platforms [2-5] in an attempt to make use of digital-image-derived data for phenotyping, which has lagged behind the vast quantities of genomic data now available [6]. To cope with the resulting surge in raw data, new tools are being developed to automate image analysis processes and assist experts in performing traditionally labour-intensive tasks such as cell scale segmentation [7,8], root length measurement [2] and architecture description [9,10]. Additionally, software tools are opening up new ways to analyse data which is not practical manually, using techniques such as optic flow [11] to examine growth at the pixel-scale, and 3D segmentation of confocal time series datasets [12].

**Figure 1:**
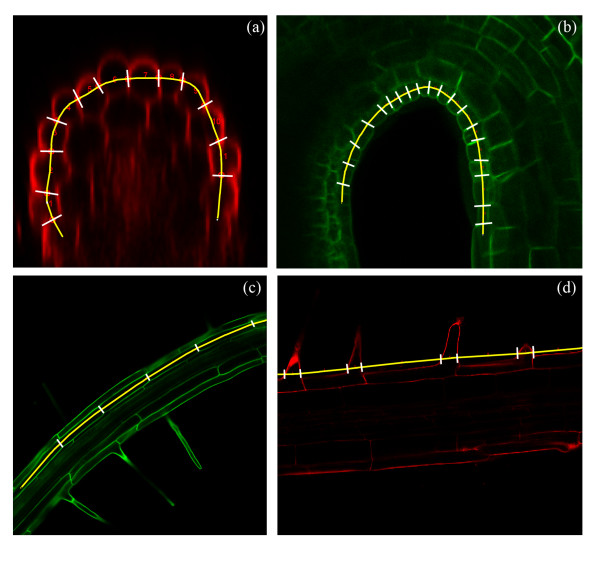
**Example uses of Cell-o-Tape**. (a) Measuring diameter of cells in propidium iodide (PI) -stained cross sections of Arabidopsis roots, (b) measuring cell lengths in an Arabidopsis hypocotyl apical hook, walls marked with GFP, (c) measuring cell lengths in a bending Arabidopsis root responding to gravity, walls marked with GFP (d) measuring parameters of emerging root hairs on a PI-stained Arabidopsis root (inter-root hair distance and width of emerging hair). Output enhanced to increase visibility

Filling a gap in the capability of such existing software, with the aims of reducing labour and decreasing subjectivity provides the motivation behind developing Cell-o-Tape, an easy-to-use software tool which semi-automates the measurement and counting of cells, and can automatically estimate the location of a feature point traditionally estimated visually. The tool has a potentially wide application area, allowing the user to measure such features on a wide range of confocal images by simply defining a profile line (Figure 1).

The tool adopts a semi-automated approach, which requires the user to define a region of interest by specifying a profile line through the cells. The user can improve the quality of the output if necessary by taking part in an iterative cycle of output refinement, adjusting parameters as necessary and even manually enhancing particularly noisy image features, although this is largely unnecessary. This semi-automatic approach, when compared to fully automatic approaches [13], has the advantage of allowing the user to define exactly where on the image the tool operates, and allows the user to improve the quality of the output using their expert knowledge, whilst performing the main labour-intensive processing in an automated, objective way.

Here, we describe the background to the problem and the algorithms used, then evaluate the success of the tool by considering a common task in root biology; identification of the transition point into the elongation zone. Cell-o-Tape's abilities to locate walls and locate this feature point are compared to results obtained by human experts working with confocal images of roots of *Arabidopsis thaliana*.

The exemplar application in this paper is the study of plant root growth, a topic which has seen increased activity as a result of the recent surge of interest in both global food security and bio-energy production. It is complex and expensive to measure plant roots at the cellular scale over time with acceptable biological replication using traditional methods, hence the requirement for automated tools.

The growth of the plant root is driven by distinct processes of cell division and cell elongation in separate developmental zones of the organ [14]. In the root meristem, cells differentiate and divide from precursor stem cells over a period of ~100 hrs. Cells then proceed through a transition zone (TSZ) before starting a process of rapid elongation in the elongation zone wherein they increase in size around 15 times over 6-8 hrs [15,16]. As root growth is driven by both the number of cells produced by cell division and the extent of their elongation, the identification of both cell size and cell number is crucial to characterisation. Existing growth analysis software typically defines kinematics of growth using pixel-scale [11,17] or cell-scale motion tracking [8,18] over image sequences, but the Cell-O-Tape approach requires only an individual image. Due to the constrained, linear growth process, a single cell-resolution image of the root contains all the growth stages and cellular scale information required, which can be thought of as a 'timeline snapshot' of these cell-scale developmental processes. Therefore, extracting cell counts and sizes from these images can provide valuable information about the underlying processes involved.

Despite the linearity of the root tissue, cell sizes in the meristem and elongation zone are notoriously heterogeneous and the measurement of a large number of cells is required to accurately characterise the nature of any growth phenotype, hence a high labour cost in performing the required measurements. Automating this process should therefore make the process more efficient and reproducible.

In addition to general cell measurements, there is one phenotypic feature of particular interest when examining cell elongation in Arabidopsis roots. The switch-point from the division zone to the elongation zone is a key observation of many growth phenotypes, usually identified as the point in the TSZ where cells start expanding rapidly, and hypothesised as the point marking the end of mitotic activity. However, there is a subjective judgement present when this point is identified by experts, as it is usually determined simply by a visual inspection of the image. In this work, as an extension to the cell-measuring capabilities of the tool and to demonstrate the accuracy and value of the cell-size data produced, we hypothesise that this point can be determined automatically and objectively from the underlying cell positions and lengths. A higher-throughput bias-free mechanism to determine this point would significantly aid growth studies. Additionally, combining the raw data and/or TSZ point calculated by Cell-o-Tape with other complimentary software kinematic approaches (eg. [19]) would provide a powerful suite of tools with which to analyse root growth.

### Implementation

**Figure 2:**
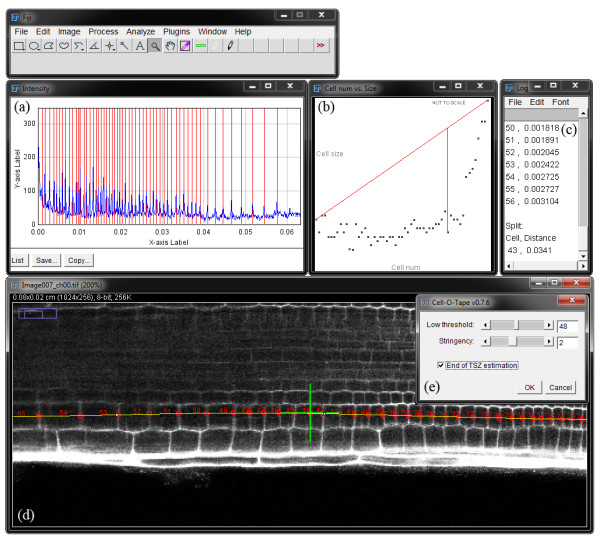
**Screenshot of the outputs of Cell-o-Tape**. (a) Intensity profile along the selection line (in blue), detected walls indicated by red vertical lines. (b) plot of cell size against cell number, with detected TSZ feature marked (*note: *the axis scaling used to clarify the display distorts the perpendicular line used to calculate this point, hence the two red lines do not appear perpendicular here, c.f. Figure 2(b-c)). (c) Text output, listing cell numbers and sizes; the TSZ feature location is also presented here, both as a cell count and distance from the cortex and endodermis initial. (d) annotated output image (e) main Cell-o-Tape dialog box for adjusting the parameters

Cell-o-Tape is implemented as a Fiji [20] macro (see Figure 2; Additional file 1), allowing biologists to use an image processing tool that they are already likely to be familiar with, as Fiji has established itself as one of the leading free, open source image analysis tools (Fiji is a fully-featured distribution of the popular ImageJ [21] software; although the tool should work within the original ImageJ, it has been developed with Fiji in mind). Macros can easily be installed in any copy of Fiji, allowing users to extend their version with the functionality of Cell-o-Tape.

Usage of the tool proceeds as follows. The approach requires a manually defined line through a cell file or group of cells, allowing the user to define which file of cells the software will measure. Once this linear region is defined using Fiji's "segmented line" tool, the macro fits a spline, producing a smooth line through the defined points. This profile line can pass through any file of cells (so long as cell walls are sufficiently clear) and be of any length; note though that to use the TSZ estimating feature which is described later, the profile line must extend sufficiently far into the elongation zone such that the change in cell length can be detected. To use this feature, ensure at least two substantially elongated cells (~4x meristem cell length) are included.

Interaction with the macro is provided at this stage, allowing the user to define a minimum intensity value expected of cell walls, to prevent false positives being detected in noisy regions of very low signal. Additionally, a slider bar is presented to set the stringency requirement (see below) of the peak detection algorithm which locates the cell walls along the defined line profile; this parameter determines how strong the intensity of the peak must be in order to be detected as a cell wall. The user can then process the image with these parameters and view the results, adjusting the parameters if necessary. The underlying algorithm is as follows (for details, see the accompanying macro code):

1. Each point along the fitted line is tested for its viability as a cell wall location. The current pixel under consideration shall be referred to as *p_t_*

2. A window of pixels of a fixed size is examined, centered on *p_t_*.

3. The minimum and maximum intensity levels (*i_min_, i_max_*) in the window are stored, ignoring *p_t_*

4. If the intensity at *p_t _*≥ *i_max _*and the intensity at *p_t _*≥ i_min _* *stringency*, then mark *p_t _*as a cell wall location.

Hypothesised wall locations are marked on the image and presented to the user. This process of user interaction followed by calculation and presentation of the results occurs in a loop so the user is able to visually refine the results until all the cell walls are detected. Empirical use of the software has shown that excellent results can be achieved this way in a minimal time on typical confocal images. See Additional file 2: Video 1 for an example of the tool in use.

The user is also presented with tools for manually enhancing walls in the image, and suppressing noise, if the data is of such low quality or contrast that they cannot all be picked out automatically. The tool is then re-run and the new walls detected.

**Figure 3:**
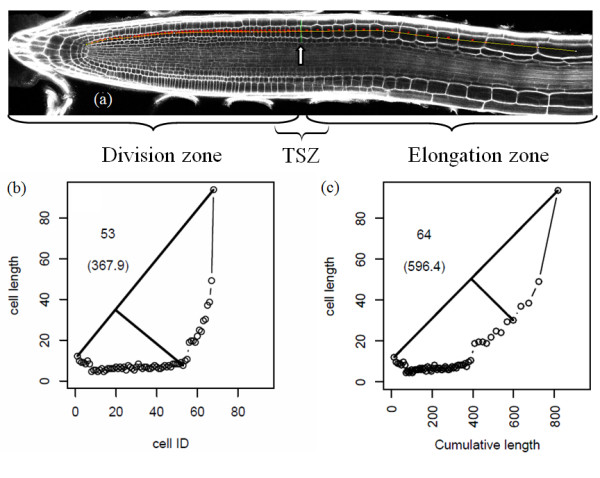
**Output image and example graphs**. (a) example output image of a measured file and predicted position of the TSZ feature (white arrow) (b) Cell number-dependent TSZ estimation of image in Figure 2a (b) cumulative length-dependent TSZ estimation

In addition to exporting raw length results and a count of cells, the tool is able to estimate the onset of an increase in cell length along the profile, such as that occurring at the division/elongation zone transition in Arabidopsis (see Figure 3(a)). This is an example of the type of further analysis that can be accomplished from the generic raw data provided by Cell-o-Tape. A geometric approach similar to that proposed in [22] was used to locate the base of the curve in cell number vs. cell length graphs (e.g. Figure 3(b); Additional file 3) and hence detect the 'break point'. By fitting a line to the first data point and the last data point (effectively the maximum as long as cells are measured into the elongation zone), and then finding the data point with the largest perpendicular distance to this line, the change-point occurring just before cell elongation begins can be found. The same approach was tested on graphs representing cumulative distance along the profile line versus cell size, but was found to be less consistent. This is due to typically less well defined curves being formed by a horizontal stretching effect introduced as the more distant cells become longer (e.g. Figure 3(c); Additional file 4); therefore this second approach was not used.

The size transition point is calculated automatically, presented on a graph and marked on the output image (Figure 3(a)); additionally the distance from the user-specified start point (in our case the cortex and endodermis initial) and cell count to this point are reported in the log window, along with all the individual cell length measures along the line (e.g. Figure 2). These heterogeneous output visualisations provide the user with multiple error checking opportunities, and additional evidence for any claims they make in publications. By saving the profile line region-of-interest from Fiji and noting the parameters for Cell-o-Tape, reviewers can re-run the algorithm on raw image data to verify claims made by authors, if necessary.

## Results and discussion

First, the accuracy of the cell length measures generated by the tool was compared to a manual approach. The detected lengths, as derived from the location of cell walls, were compared to manual measures of length. A root-mean-square error of 0.3 μm (*n *= 66 cells, length range ~10-90 μm) shows a good agreement with groundtruth manual length measures.

Second, the ability of the tool to predict the position of the transition into the elongation zone in roots of Arabidopsis was tested by comparing the results to experts in the field. Plant biologists experienced in estimating the end of TSZ landmark location were asked to locate this point by eye on a set of 26 images and mark its position, and the resulting distance and cell count to this point from the cortex and endodermis initial were recorded manually by measuring the marked image. Cell-o-Tape was used for the same images and the results compared. Identifying a result as 'correct' is problematic in scenarios such as this, as it is not clear if the error lies with the expert's subjective ground truth, or the new software. Therefore, we wish to know simply if the new tool can be expected to agree with the group of experts as well as they agree with each other. To this end, we calculate the Williams Index, which computes a ratio between the average computer-to-expert agreement and the average inter-expert agreement [23,24], and provides a measure of the significance of the agreement:

(1) $$I^{\prime }=\frac{\frac{1}{n}\sum_{j=1}^{n}\frac{1}{Do,j}}{\frac{2}{n(n-1)}\sum_{j}\sum_{j^{\prime }:j^{\prime }>j}\frac{1}{D_{j,j^{\prime }}}}$$

**Figure 4:**
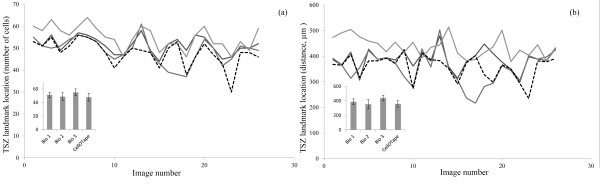
**Graphs illustrating the variation in TSZ landmark estimation**. Graphs illustrating the variation in TSZ landmark estimation between human experts (solid grey) and the software (dotted), where location is defined as cell number (**a**) and distance along the root (**b**), *n *26 images. Inset charts show average measurements for each expert and the software across all images (bars represent 1 S.D)

Reporting the TSZ landmark location in terms of distance along the root yielded an *I' *of 1.31; reporting in terms of number of cells gave *I' *= 1.01. When interpreting the Williams Index, if the limit of the upper confidence interval (CI) of *I' *is greater than 1, then we can infer that the computer generated estimation agrees with the expert estimations as much as the expert estimations agree with one another [23]. Here, in both cases *I' *> 1, so computation of the CI is not necessary as it will clearly exceed 1. Hence both the distance- and cell count-reporting variations of Cell-o-Tape provide estimates of the TSZ landmark location which agree with the expert estimations as much as the expert estimations agree with each another. The accuracy and variability in this data is also shown graphically in Figure 4.

To assess how sensitive the tool is to how far the profile line extends along the root when calculating the TSZ point, two variations were considered. In the first case, a situation where the user does not extend the profile line far enough into the elongation zone was considered. To test this, the TSZ point was recalculated for the 26 images, but discarding the final two measurements along each profile line (typically the two largest cells); see Additional file 5. On 18 occasions (70%) this made no difference to the TSZ point estimation. On the 8 images where the location of the point differed, the average error in cell location number was 7.5 cells (15%). So, while on most occasions no extra error is introduced if the profile line is terminated too early, there is a chance of a significant error. On examining the cell ID versus cell length graphs, this error is likely being caused by the graphs becoming overly compressed on the vertical axis, caused by the omission of the longer cells, therefore invalidating the assumption of a detectable corner point [22] as well as making the technique more sensitive to noise. Therefore, it is recommended that the profile line be extended to include at least two substantially elongated cells (~4x meristem cell length). This would seem reasonable, as any approach that estimates a feature such as the TSZ point would need a sufficiently extensive data set as a starting point.

The complementary situation, where the profile line extends further towards the mature zone, was also examined. This was achieved by defining the profile line further towards the mature zone on a composite image (Additional file 6). It was expected that this case would not cause problems for the calculation of the TSZ point as there should still be a detectable corner point on the graph [22]. As can be seen in Additional file 6, the TSZ is still located correctly on this longer profile line, and the more elongated cells still correctly delineated.

## Conclusions

We have presented Cell-o-Tape, a tool primarily designed for automatically counting and measuring cells along a defined profile in confocal images. Cell-o-Tape can also identify a significant change in cell length along the profile, a facility which can be used to estimate the location of the start of rapid cell elongation along a file of cells in the Arabidopsis root, as shown in our example application.

The tool was found to be able to accurately measure cells compared to a human expert. A method was presented to determine the location of a TSZ feature point by detecting a 'break point' on a cell number versus cell size graph. This process was found to estimate the position of this TSZ landmark point in agreement with manual expert measures as well as the experts agree with each other. In addition, reporting the location of the TSZ in terms of distance along the root yielded a higher Williams Index than when the number of cells was used as the comparison metric, although this may be due to the higher variability between the experts in this case (Figure 4). Additionally, it was observed that the experts sometimes agreed on the TSZ landmark location using distance, but provided a differing cell count. This highlights the subjective nature of counting cells, both in terms of simple miscounting, and misinterpreting noise or 'ghosting' from surrounding cell layers as erroneous cell walls; these factors are not present when reporting the location in terms of distance along the root. Thus reporting the TSZ location using distance would seem to be a more reliable measure in general than providing a cell count, as it is not as sensitive to missed cells. In both cases, the tool is seen to agree with the experts, and thus provides an objective and reproducible way to perform these measurements.

We recommend using the tool as an efficient way to measure cell lengths and count cells along cell files with minimum interaction required by the user, and also to provide an impartial estimate for the TSZ feature location in Arabidopsis roots, alongside the user's expert but subjective opinion. The graph the tool presents can also be used to support this location, and this representation also helps alert the user to artefacts in the process, meaning they can then re-examine the image if necessary. Although developed for Arabidopsis root images, we see no reason why the tool could not be used on other confocal images with similar content.

### Availability and requirements

The tool is open source, and available from the CPIB website, and requires Fiji [20]. Reviewed version is also supplied as Additional file 1.

**Project name: **Cell-o-Tape

**Project home page: **http://www.cpib.ac.uk/software/

**Operating system(s): **Any Imagej/Fiji supports

**Programming language: **ImageJ/Fiji macro language

**Other requirements: **Developed on Fiji 1.45r

**License: **BSD.

## Competing interests

The authors declare that they have no competing interests.

## Authors' contributions

APF implemented an enhanced version of MHW's original peak finding approach as a Fiji plugin. APF, DMW and TPP drafted the paper, and DMW provided initial testing. KK provided mathematical advice and algorithms for finding the 'break point' on the growth curves. DD captured the image data and provided analysis; further analysis was carried out by UV and SUT. All authors read and commented on the paper.

## Supplementary Material

Additional file 1 **Cell-o-tape.zip. Fiji macro file, example image and quickstart guide**. Click here for file

Additional file 2 **Cell-o-tape_demo.wmv. Screen capture video of the software being used**. Click here for file

Additional file 3 **Cell Length vs cell ID.pdf**. Location of TSZ feature calculated on each image graph using cell number. Click here for file

Additional file 4 **Cell Length vs cumulative length.pdf**. Location of TSZ feature calculated on each image graph using cumulative length. Click here for file

Additional file 5 **Cell Length vs cell ID ignore2points.pdf**. Location of TSZ point calculated on each image graph using cell number, and discounting the final 2 points to simulate a profile line which is shorter than expected. Click here for file

Additional file 6 **Long profile.png**. Example output image where the profile line is extended further along the root towards the mature zone. The TSZ point is still correctly calculated and the longer cells correctly identified. Click here for file
