# Supplementary figures and images for: Identifying biological landmarks using a novel cell measuring image analysis tool: Cell-o-Tape
Source: Plant Methods. 2012 Mar 2;8:7. doi: 10.1186/1746-4811-8-7 (PMC3359173; doi:10.1186/1746-4811-8-7)

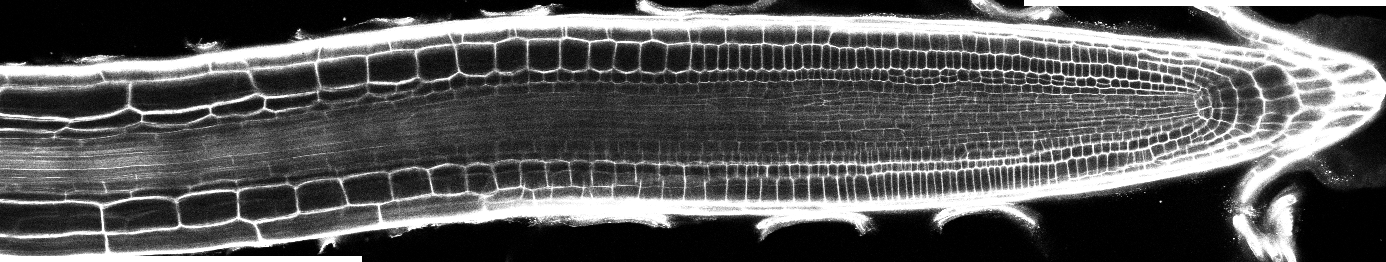

Supplement: Additional file 1 — Cell-o-tape.zip. Fiji macro file, example image and quickstart guide. [file 1746-4811-8-7-S1.ZIP › example_input.tif]

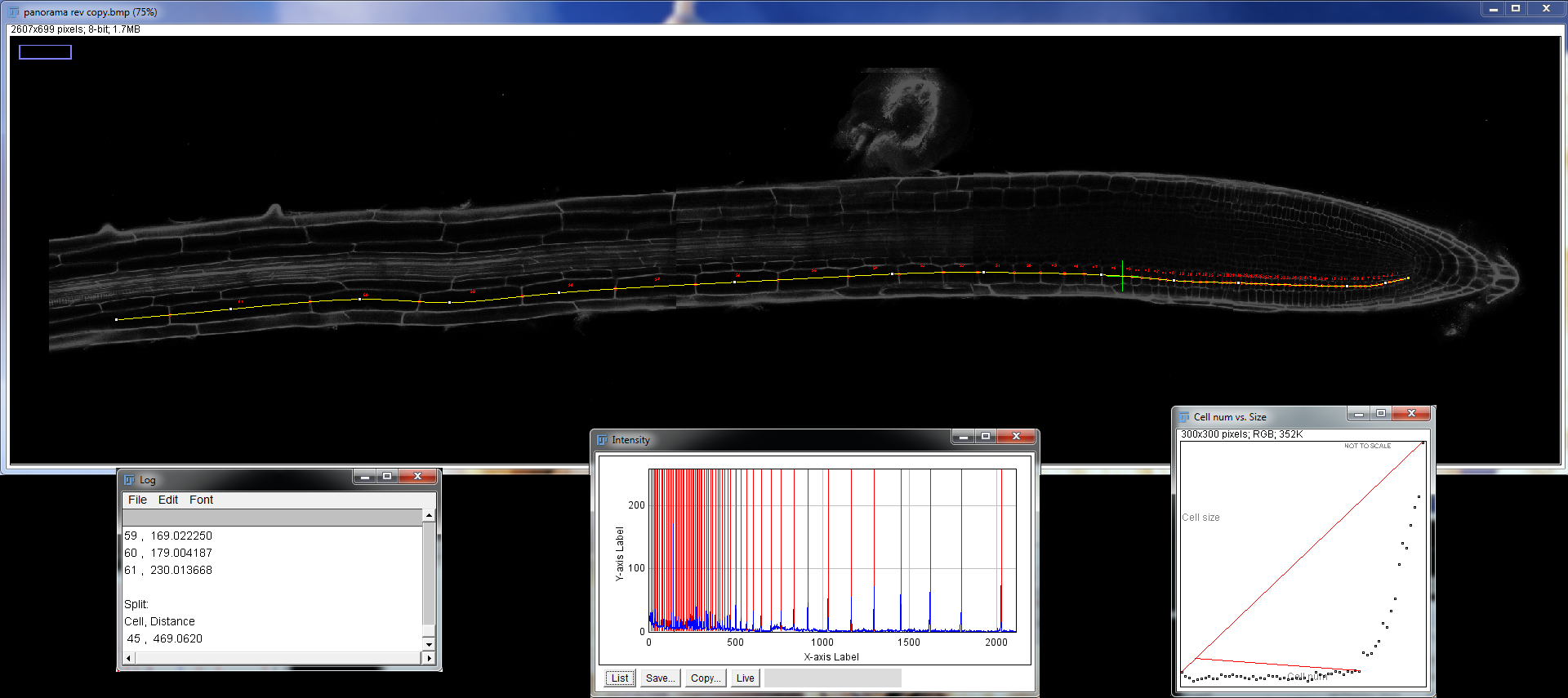

Supplement: Additional file 6 — Long profile.png. Example output image where the profile line is extended further along the root towards the mature zone. The TSZ point is still correctly calculated and the longer cells correctly identified. [file 1746-4811-8-7-S6.PNG]
