# Supplementary material for: Identifying biological landmarks using a novel cell measuring image analysis tool: Cell-o-Tape
Source: Plant Methods. 2012 Mar 2;8:7. doi: 10.1186/1746-4811-8-7 (PMC3359173; doi:10.1186/1746-4811-8-7)
Supplement: Additional file 3 — Cell Length vs cell ID.pdf. Location of TSZ feature calculated on each image graph using cell number. [file 1746-4811-8-7-S3.PDF]

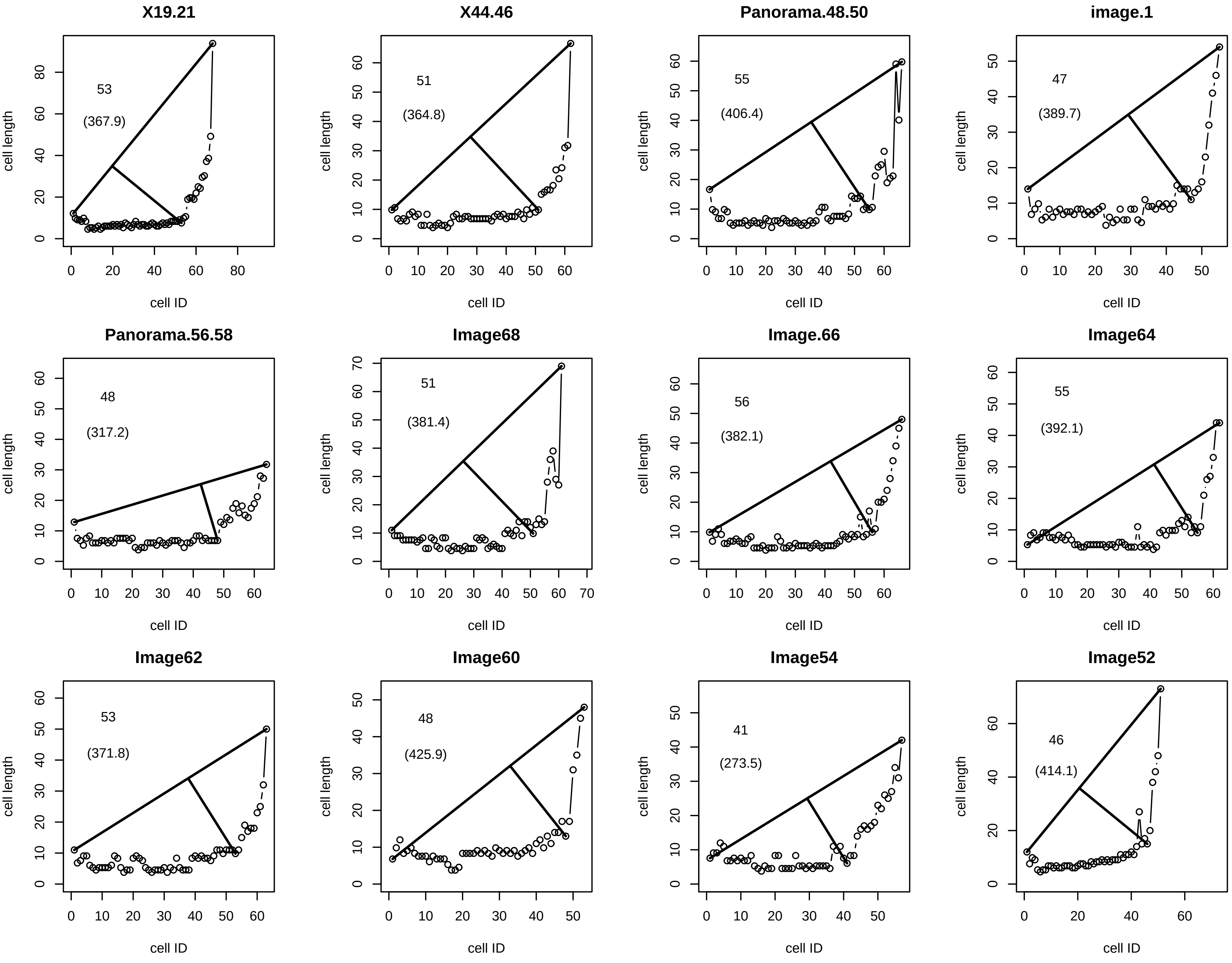
X19.21
53
(367.9)
X44.46
51
(364.8)
Panorama.48.50
55
(406.4)
image.1
47
(389.7)
Panorama.56.58
48
(317.2)
Image68
51
(381.4)
Image.66
56
(382.1)
Image64
55
(392.1)
Image62
53
(371.8)
Image60
48
(425.9)
Image54
41
(273.5)
Image52
46
(414.1)
cell length
cell ID

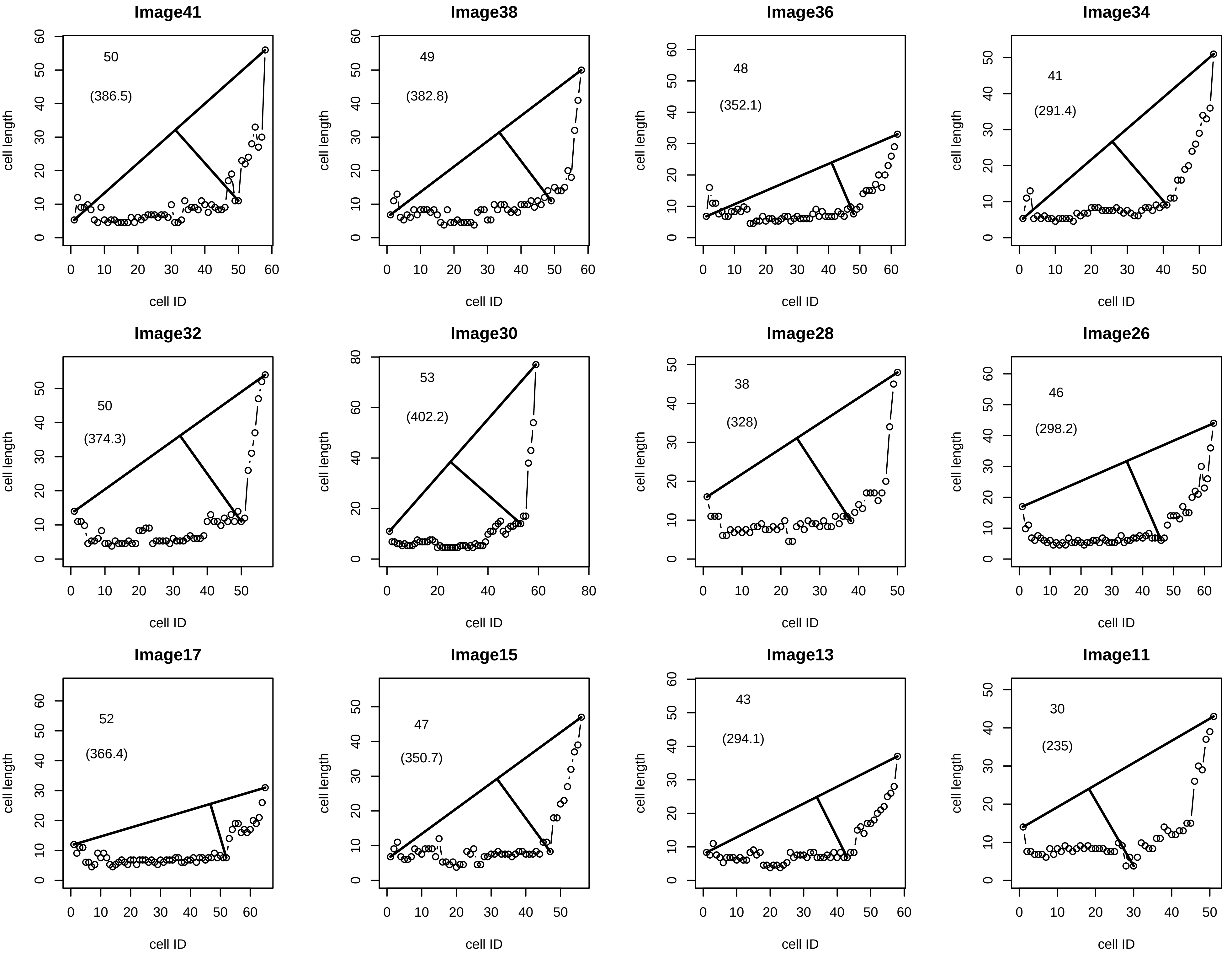
Image41
50
(386.5)
Image38
49
(382.8)
Image36
48
(352.1)
Image34
41
(291.4)
Image32
50
(374.3)
Image30
53
(402.2)
Image28
38
(328)
Image26
46
(298.2)
Image17
52
(366.4)
Image15
47
(350.7)
Image13
43
(294.1)
Image11
30
(235)
cell length
cell ID

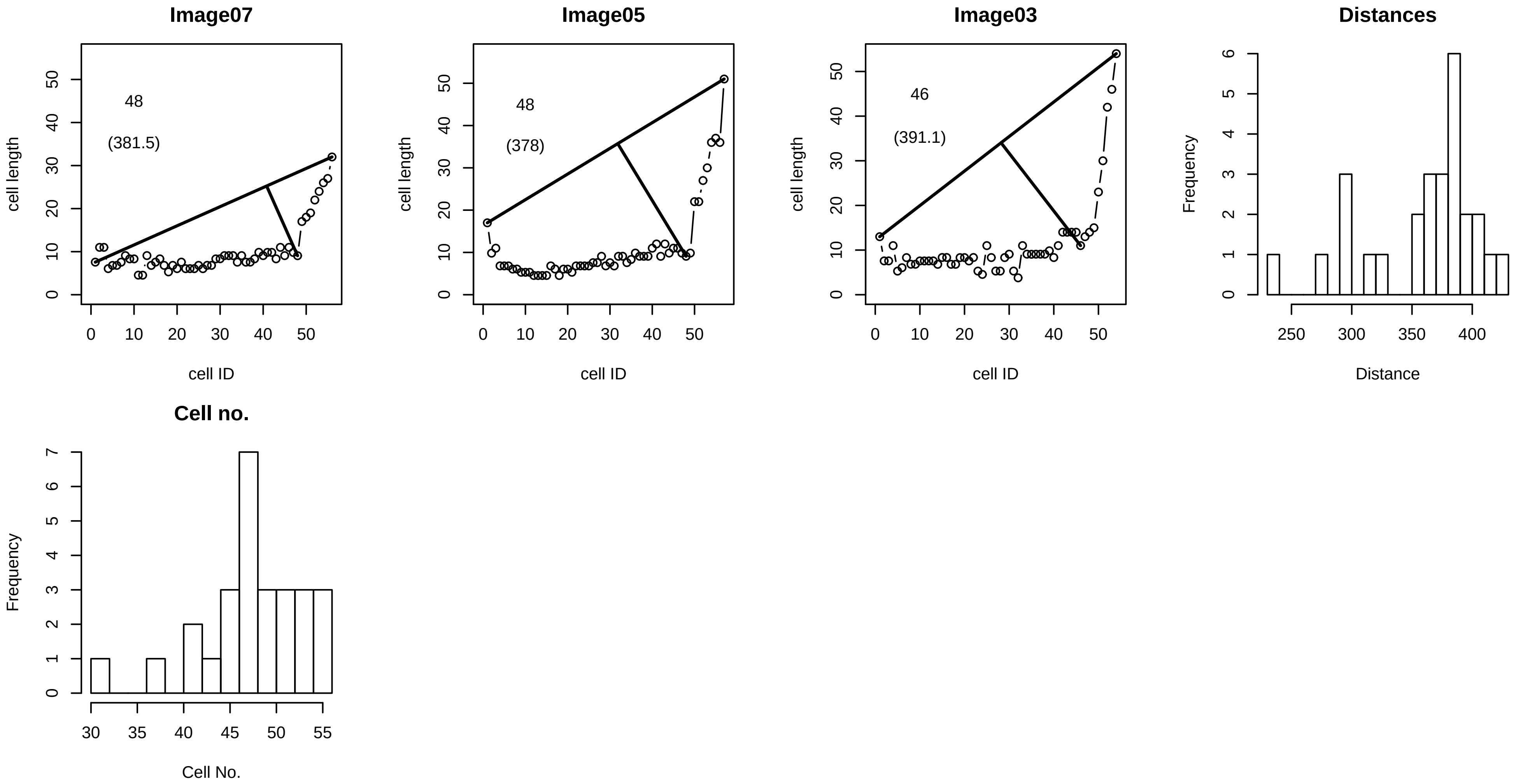
Image07
48
(381.5)
cell length
cell ID
Image05
48
(378)
cell length
cell ID
Image03
46
(391.1)
cell length
cell ID
Distances
Frequency
Distance
Cell no.
Frequency
Cell No.
