# Supplementary material for: Identifying biological landmarks using a novel cell measuring image analysis tool: Cell-o-Tape
Source: Plant Methods. 2012 Mar 2;8:7. doi: 10.1186/1746-4811-8-7 (PMC3359173; doi:10.1186/1746-4811-8-7)
Supplement: Additional file 4 — Cell Length vs cumulative length.pdf. Location of TSZ feature calculated on each image graph using cumulative length. [file 1746-4811-8-7-S4.PDF]

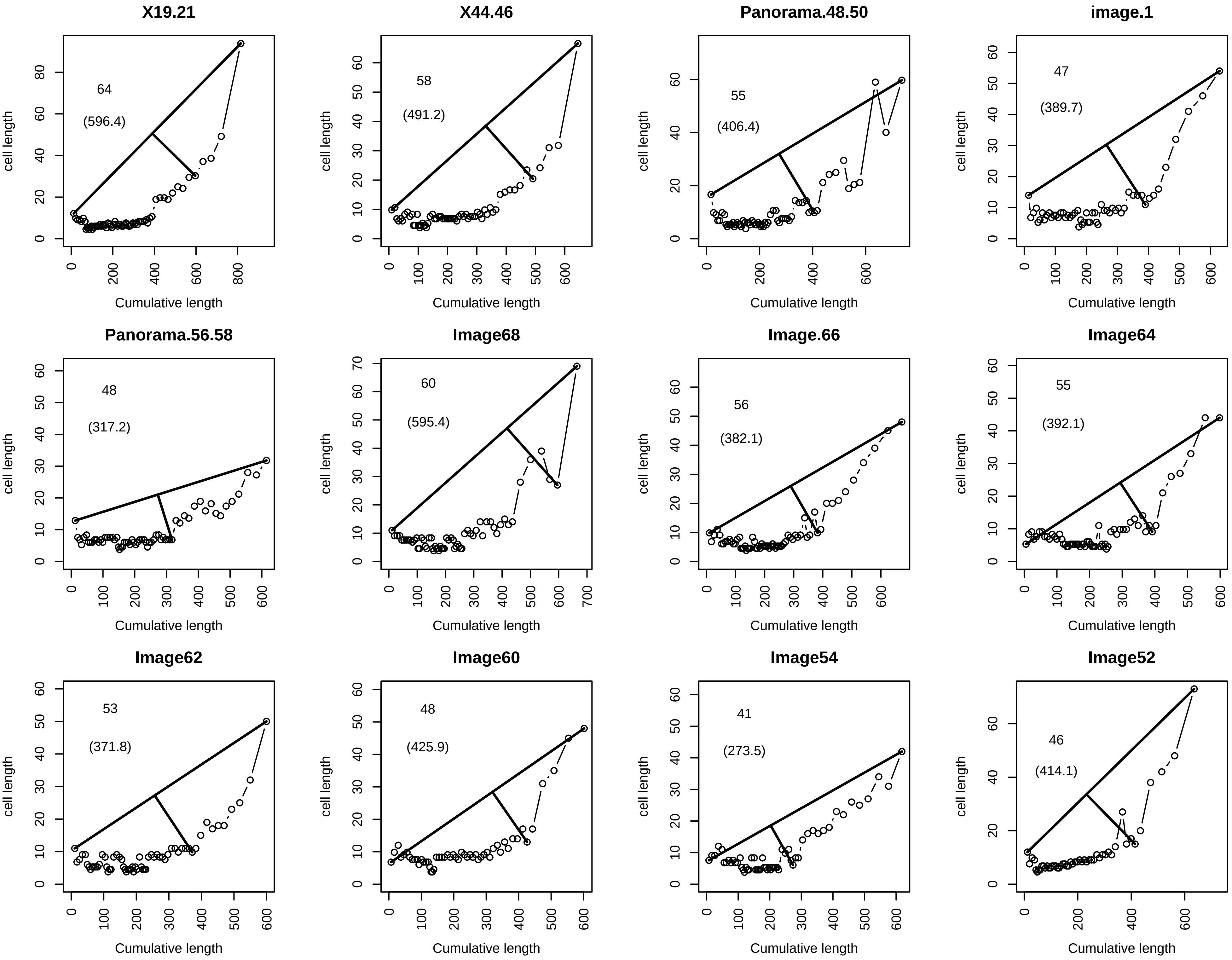
X19.21
64
(596.4)
X44.46
58
(491.2)
Panorama.48.50
55
(406.4)
image.1
47
(389.7)
Panorama.56.58
48
(317.2)
Image68
60
(595.4)
Image.66
56
(382.1)
Image64
55
(392.1)
Image62
53
(371.8)
Image60
48
(425.9)
Image54
41
(273.5)
Image52
46
(414.1)
cell length
Cumulative length

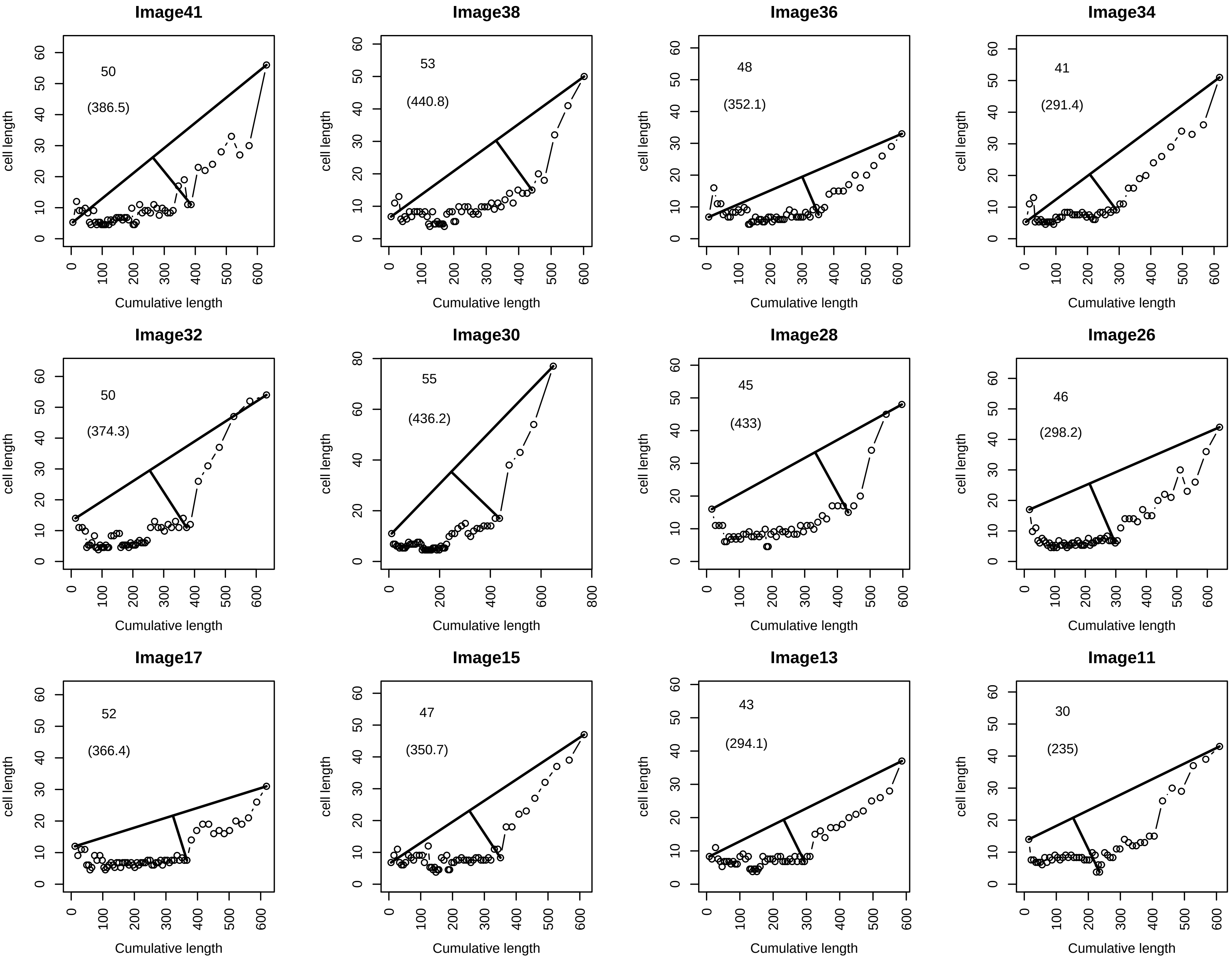
Image41
50
(386.5)
Image38
53
(440.8)
Image36
48
(352.1)
Image34
41
(291.4)
Image32
50
(374.3)
Image30
55
(436.2)
Image28
45
(433)
Image26
46
(298.2)
Image17
52
(366.4)
Image15
47
(350.7)
Image13
43
(294.1)
Image11
30
(235)
cell length
Cumulative length

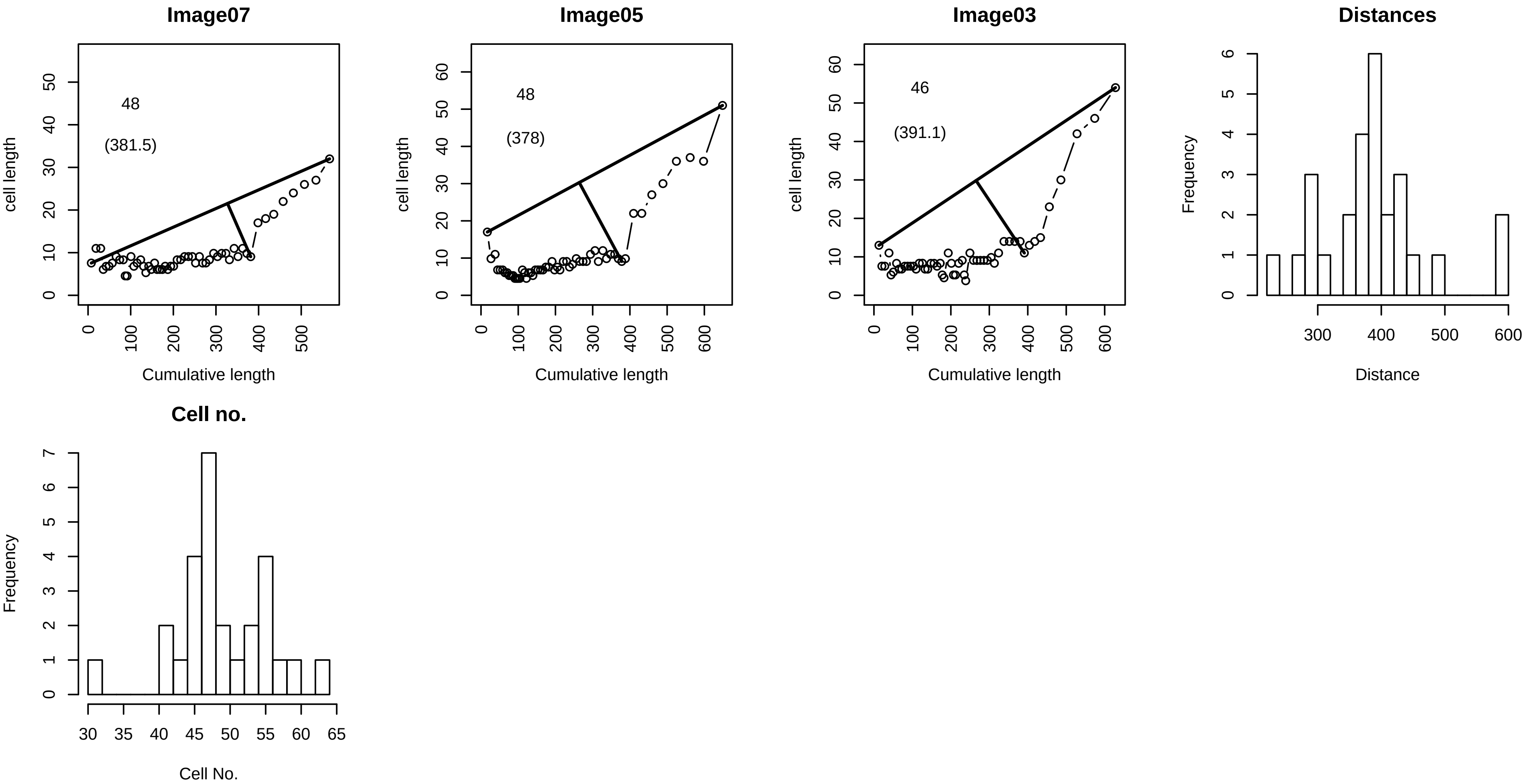
Image07
48
(381.5)
cell length
Cumulative length
Image05
48
(378)
cell length
Cumulative length
Image03
46
(391.1)
cell length
Cumulative length
Distances
Frequency
Distance
Cell no.
Frequency
Cell No.
