# Supplementary material for: Identifying biological landmarks using a novel cell measuring image analysis tool: Cell-o-Tape
Source: Plant Methods. 2012 Mar 2;8:7. doi: 10.1186/1746-4811-8-7 (PMC3359173; doi:10.1186/1746-4811-8-7)
Supplement: Additional file 5 — Cell Length vs cell ID ignore2points.pdf. Location of TSZ point calculated on each image graph using cell number, and discounting the final 2 points to simulate a profile line which is shorter than expected. [file 1746-4811-8-7-S5.PDF]

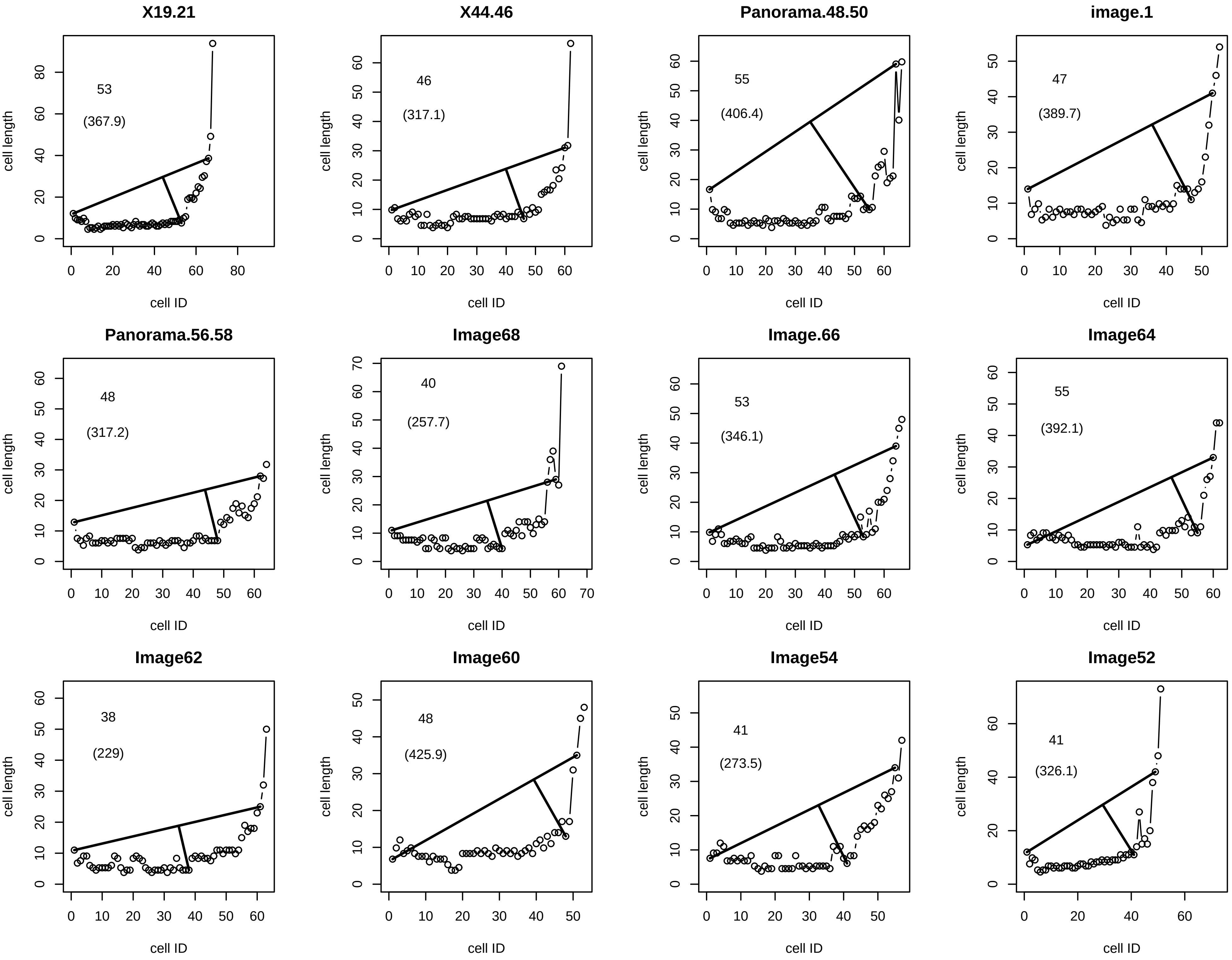

X19.21
53
(367.9)
X44.46
46
(317.1)
Panorama.48.50
55
(406.4)
image.1
47
(389.7)
Panorama.56.58
48
(317.2)
Image68
40
(257.7)
Image.66
53
(346.1)
Image64
55
(392.1)
Image62
38
(229)
Image60
48
(425.9)
Image54
41
(273.5)
Image52
41
(326.1)
cell length
cell ID

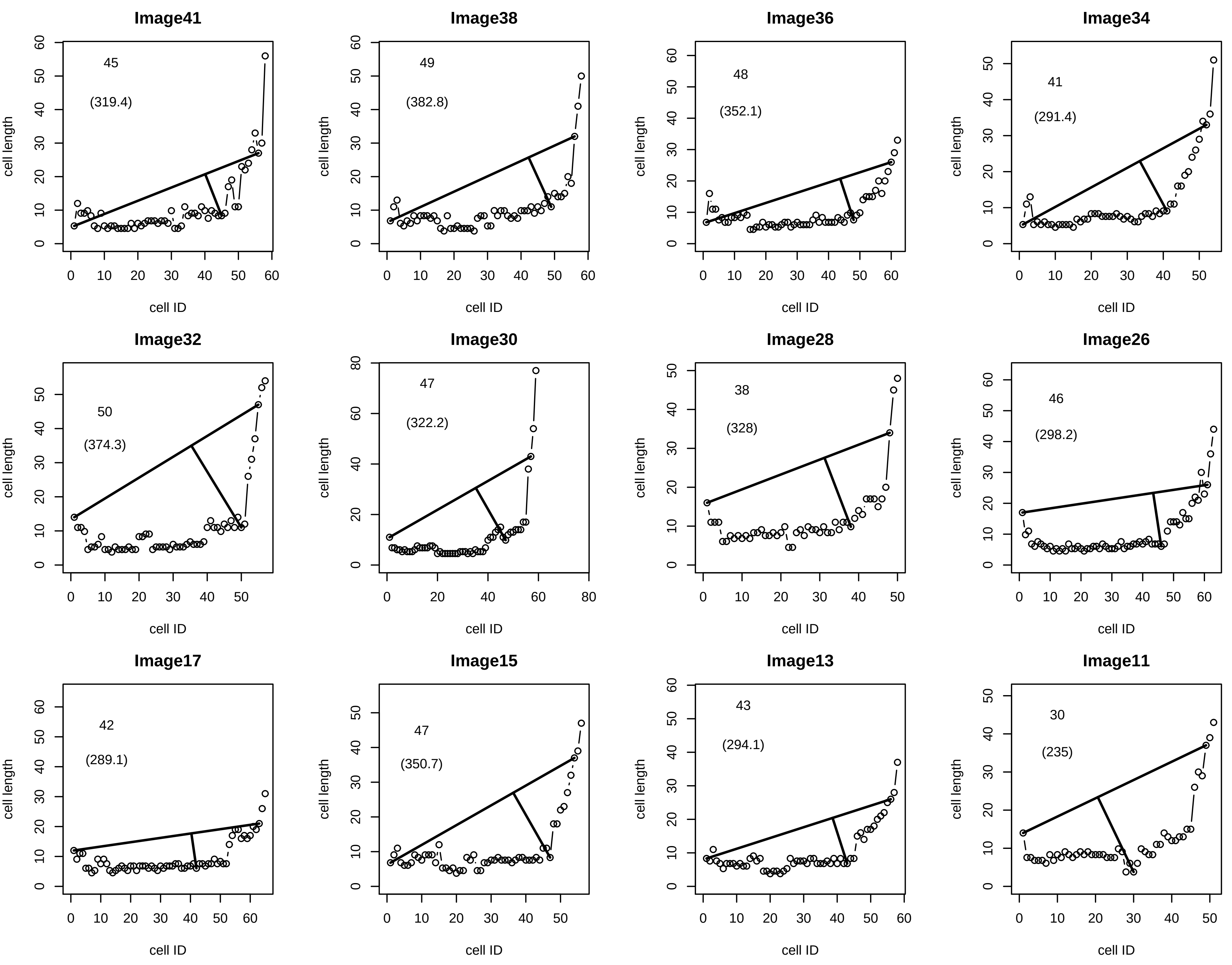
Image41
45
(319.4)
Image38
49
(382.8)
Image36
48
(352.1)
Image34
41
(291.4)
Image32
50
(374.3)
Image30
47
(322.2)
Image28
38
(328)
Image26
46
(298.2)
Image17
42
(289.1)
Image15
47
(350.7)
Image13
43
(294.1)
Image11
30
(235)
cell length
cell ID

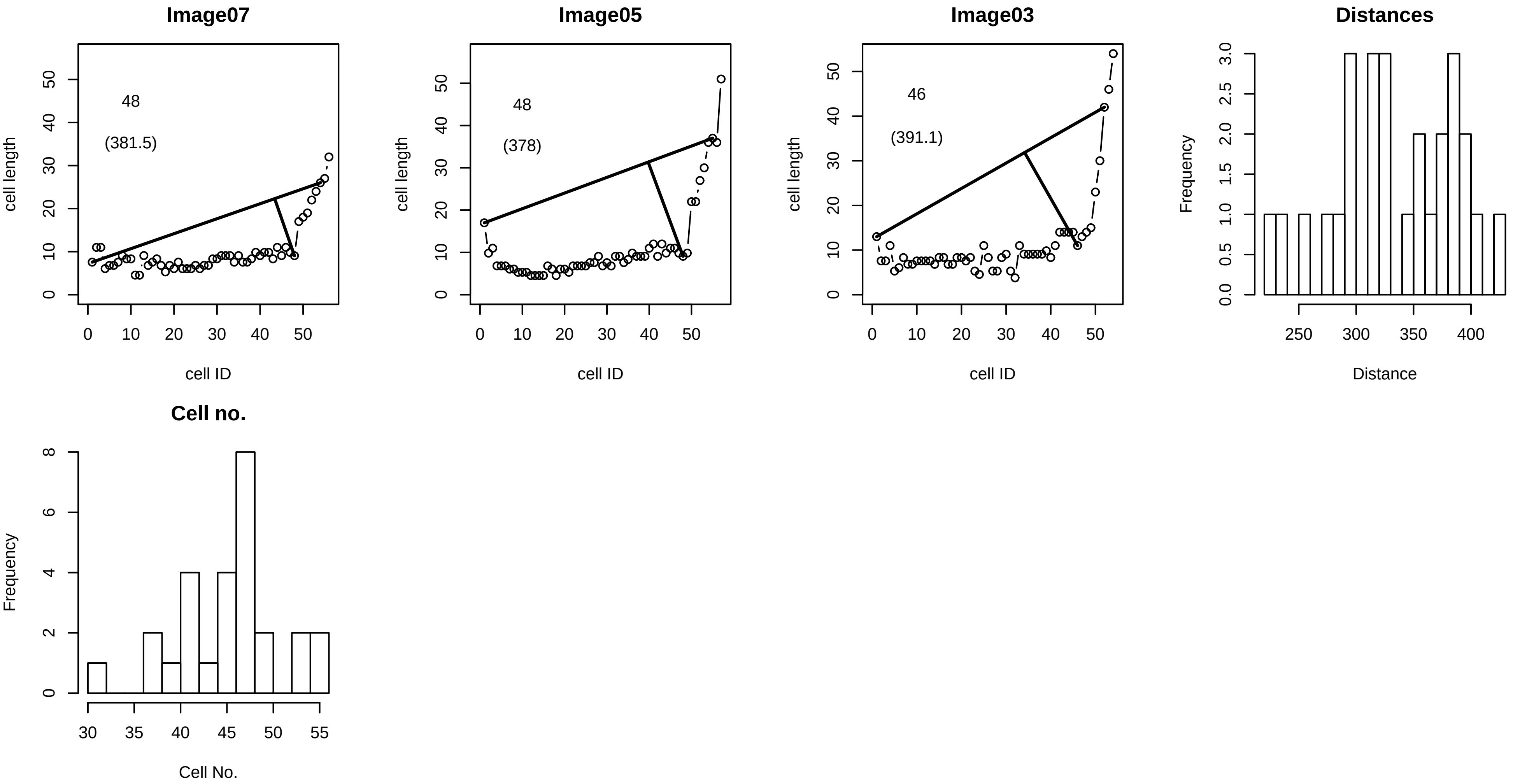
Image07
48
(381.5)
cell length
cell ID
Image05
48
(378)
cell length
cell ID
Image03
46
(391.1)
cell length
cell ID
Distances
Frequency
Distance
Cell no.
Frequency
Cell No.
